# Predictive factors and treatment challenges in malignant progression of relapsing-remitting multiple sclerosis

**DOI:** 10.1016/j.heliyon.2024.e26658

**Published:** 2024-02-17

**Authors:** Masoud Ghiasian, Rashed Bawand, Sulmaz Jabarzadeh, Abbas Moradi

**Affiliations:** a Department of Neuroimmunology, School of Medicine, Hamadan University of Medical Sciences, Hamadan, Iran; b Department of General Medicine, School of Medicine, Hamadan University of Medical Sciences, Hamadan, Iran; c Department of Neurology, School of Medicine, Hamadan University of Medical Sciences, Hamadan, Iran; d Department of Social Medicine, School of Medicine, Hamadan University of Medical Sciences, Hamadan, Iran

**Keywords:** Relapsing-Remitting Multiple Sclerosis (RRMS), Prognosis, Malignant MS, Benign MS, Escalation, Disease-modifying therapies

## Abstract

**Objective:**

Our objective was to uncover the predictive factors that can help anticipate the malignant progression of individuals with Relapsing-Remitting Multiple Sclerosis (RRMS). Additionally, we sought to analyze and compare the response to treatment between patients with benign and malignant forms of RRMS.

**Methods:**

This cohort study included RRMS patients categorized as benign (≥10 years since disease onset, Expanded Disability Status Scale (EDSS) ≤ 1) or malignant (≤5 years since disease onset, EDSS ≥6). Patients’ data, including demographics, medical history, treatment, and MRI (Magnetic Resonance Imaging) scans, were collected and statistically analyzed.

**Results:**

Among the 254 patients diagnosed with RRMS, 174 were found to have benign RRMS, while the remaining 80 were diagnosed with malignant RRMS. Notably, patients with malignant RRMS exhibited a significantly higher mean age of onset (32.00 ± 7.96 vs. 25.70 ± 17.19; *P* < 0.001) and a greater prevalence of males (40% vs. 18.4%; *P* = 0.014*)*. Additionally, within the initial five years of diagnosis, patients with malignant RRMS experienced a higher number of relapses (median: 4 vs. 2; *P* < 0.001) and hospitalizations (median: 2 vs. 1; *P* = 0.006) compared to those with benign RRMS. Clinical presentations of malignant RRMS were predominantly characterized by multifocal attacks, whereas unifocal attacks were more prevalent in patients with benign RRMS. MRI scans revealed that malignant RRMS patients displayed a higher burden of plaques in the infratentorial and cord regions, as well as a greater number of black hole lesions. Conversely, benign RRMS patients exhibited a higher number of Gadolinium-enhanced lesions. Utilizing Disease-Modifying Therapies (DMTs) with an escalating approach has shown effectiveness in managing benign RRMS. However, it has proven insufficient in addressing malignant RRMS, resulting in frequent transitions to higher-line DMTs. As a result, it places a considerable burden on patients with malignant RRMS, consuming valuable time and resources, and ultimately yielding subpar outcomes.

**Conclusion:**

Our study identifies prognostic factors for malignant progression in RRMS, including older age of onset, male gender, increased relapses and hospitalizations, multifocal attacks, higher plaque load, and black hole lesions. The current escalation strategy for DMTs is insufficient for managing malignant RRMS, requiring alternative approaches for improved outcomes. In other words, MS is a spectrum rather than a single disease, and some patients progress to a malignant phenotype of MS that is not effectively treated by the current approach.

## 1 Introduction

Multiple Sclerosis (MS) is a chronic and progressive disorder of the Central Nervous System (CNS) that can result in severe disability for patients and have significant social and economic consequences [1]. Among CNS disorders, MS is the leading cause of permanent disability in young adults, second only to trauma [2]. This autoimmune disease is associated with inflammation and myelin sheath involvement in the CNS and has a varied clinical spectrum [1]. Patients with MS typically present with a variety of clinical symptoms, including but not limited to sensory disturbances, vision impairments, movement disorders, issues with walking and balance, dizziness, sphincteric problems, transverse myelitis, pain, etc. [1]. The progression of the disease is unpredictable and can vary from person to person, indicating the involvement of different nerves and the diverse process of disease progression [1].

There are several types of MS, and the classification of MS is based on the course of the disease. The most common type of MS is relapsing-remitting MS (RRMS), which accounts for approximately 85% of cases. RRMS is characterized by distinct episodes of symptom exacerbation (relapse) followed by periods of partial or complete recovery (remission). Other types of MS include primary progressive MS (PPMS), secondary progressive MS (SPMS), and progressive-relapsing MS (PRMS). Each type of MS has different clinical features and treatment options. RRMS is the most studied and treatable form of MS, and early intervention can significantly delay disease progression and improve patient outcomes [3].

Although great individual variability exists in disease prognosis, a variety of probable factors (such as age, sex, initial disease course, initial manifestations, etc.) have been identified as possible prognostic indicators [1]. Research shows that about 17% of patients with MS for at least a decade show mild or no disability; however, some individuals may experience significant disability soon after symptom onset [4]. Therefore, identifying prognostic markers plays a vital role in forecasting the potential progression of MS towards a more aggressive form. By recognizing these markers early, it becomes possible to commence suitable treatment promptly. This early intervention is key to averting complications and adverse outcomes, and it can substantially decelerate the disease's progression.

The primary objective of this study was to methodically identify and evaluate predictive factors that significantly influence the progression of RRMS. Specifically, our research aimed to discern the clinical, demographic, and MRI characteristics that differentiate patients who maintain a benign course of RRMS over a decade from those who advance towards a malignant phenotype within a span of five years. Additionally, we sought to critically analyze the impact of various Disease-Modifying Therapies (DMTs) and treatment approaches, particularly focusing on the effectiveness of the escalation strategy in managing benign versus malignant MS cases. The ultimate goal of this research was to provide a comprehensive understanding of the factors influencing RRMS progression and to identify reliable prognostic markers. These markers could significantly aid healthcare professionals in promptly selecting the most appropriate and effective treatment strategies, ultimately leading to improved patient outcomes and quality of life in those affected by this challenging and unpredictable disease.

## 2 Materials & methods

### 2.1 General principles

This is an observational retrospective cohort study, which has received approval from the Ethics committee (Code. IR.UMSHA.REC.1400.831) and the Institutional Review Board (IRB) (No. 140011199613) at the Hamadan University of Medical Sciences. Informed consent was obtained from all participants in accordance with the Declaration of Helsinki. Participants were free to withdraw from the study at any time, and their decision did not impact their medical care. Data were collected anonymously and the results were not disclosed to any individual or organization.

### 2.2 Patients

The study used a consecutive available sampling method to evaluate samples from eligible volunteers with RRMS who were continuously monitored and treated since their diagnosis at Shahid Beheshti Hospital and/or Neshat Clinic in Hamadan, Iran, and could be categorized as having benign or malignant MS. In other words, we specifically compared two distinct groups of RRMS patients: those who maintained minimal disability over a prolonged period and those who developed severe disabilities relatively quickly. By intentionally excluding patients who fell in the middle of the disability spectrum, we aimed to contrast the extreme ends of the spectrum more clearly. This approach allowed us to better evaluate and understand the prognostic factors influencing the diverse progression paths in RRMS. The patients' medical records and information were transparent and easily accessible. To participate in the study, patients had to receive standard DMT treatment based on FDA-approved indications, and those with chronic diseases other than MS were excluded.

### 2.3 Proceedings

Following the acquisition of informed consent from the patients, a thorough evaluation of their files and medical records was conducted. The accuracy of the contents was further confirmed through interviews and direct questions. The extracted information encompassed several key factors, including the patients' age at the time of diagnosis, their gender, familial history of MS, number of relapses, frequency of hospitalizations, and adherence to DMTs. Additionally, the type of DMTs administered, as well as any changes in treatment during the course of the disease, were carefully documented.

Furthermore, the initial presentation and relapses of MS were evaluated to determine the nature of attacks, which were then categorized as either unifocal, involving symptoms related to sensation, motor function, vision, balance, etc. or multifocal.

The MRI scans taken at the time of diagnosis (initial MRIs), as well as the final scans taken after 5 years for malignant cases and 10 years for benign cases (final MRIs), were assessed. It is important to mention that the study did not incorporate the consecutive MRIs that were conducted throughout the progression of the illness. The analysis focused on the quantity and location of MS plaques, including those found in the supratentorial, infratentorial (brain stem & cerebellum), and cord regions. Additionally, the scans were evaluated for the presence of Gadolinium absorption (Gad-enhancement) and lesions known as black holes. A standardized approach was adopted for MRI examinations of all patients, with and without contrast, utilizing a 1.5 T MRI machine located at Shahid Beheshti Hospital in Hamedan. Furthermore, initial and final MRIs were available for all the included patients, and the analysis of all MRI scans was performed by a solitary radiologist to ensure consistency and accuracy.

### 2.4 Definitions

✓ *Expanded Disability Status Scale (EDSS):* It is a scale used for assessing and quantifying the level of disability in patients with MS, and monitoring changes in the level of disability over time. It is a scale that ranges from 0 to 10 in half-point increments, with higher scores indicating greater disability. The EDSS evaluates multiple functional systems to determine the patient's level of disability, including Visual functions, Brainstem functions, Pyramidal functions (motor), Cerebellar functions (coordination and balance), Sensory functions, Bowel and Bladder functions, Cerebral (mental) functions, and Ambulation (walking ability). This comprehensive assessment is crucial in evaluating the overall impact of MS on an individual's daily life and functioning, making it a widely used and functional measure in the management and study of MS [5].
✓ *Benign MS:* Refers to patients who have had MS for at least 10 years with an EDSS score of 1 or less (While there are multiple definitions of benign MS present in the literature [4,[6], [7], [8], [9], [10], [11], [12], [13], [14], [15], [16], [17], [18], [19], [20]], a comprehensive agreement has yet to be reached. However, the majority of studies have defined benign MS as having an EDSS score of less than 3 within 10 years of disease onset [6,[13], [14], [15],[17], [18], [19], [20]]. Therefore, to ensure greater caution and validity in our findings, we only considered patients with an EDSS score of 1 or less after at least 10 years of disease onset as having benign MS.).
✓ *Malignant MS:* Refers to patients who have MS within the last 5 years and have an EDSS score of 6 or higher [[21], [22], [23]].
✓ *MS MRI plaques:* MS MRI plaque is a visible area of damage or inflammation on the brain or spinal cord MRI caused by an abnormal immune response that targets and damages the myelin. Radiologic characteristics of MS MRI plaques include hyperintense ovoid lesions with a diameter larger than 3 mm. This study utilized a comprehensive approach to detect the presence of plaques. MRIs were evaluated in all three axial, coronal, and sagittal sections to ensure accuracy. Only lesions that were identified in all three sections were considered as plaques. To detect supratentorial plaques, the FLAIR (Fluid attenuated inversion recovery) sequence was used, while T2-weighted was used to examine infratentorial plaques. Finally, the proton density-weighted sequence was utilized to detect cord plaques, ensuring that all types of plaques were properly identified.
✓ *Black hole lesion:* The condition in which, MS lesions evolve into persistent hypointense lesions on T1-weighted MRI is known as “black holes”, which can represent stable lesions where severe tissue disruption has occurred [24].
✓ *MS attack:* Patients who developed new MS-related signs & symptoms that lasted more than 24 h and were not secondary to fever or infection were considered to have an MS attack (or clinical relapse) [1].
✓ *DMT selection & changes:* Established guidelines classify DMTs into three lines based on their potency and potential adverse effects. The first line of treatment consists of drugs like Interferons, Dimethyl fumarate, Glatiramer acetate, Teriflunomide, and others. Second-line therapies include Fingolimod, Natalizumab, Rituximab, Ocrelizumab, and similar drugs. The third and final line of DMTs consists of more potent drugs such as Mitoxantrone and Cyclophosphamide. There are three different therapeutic approaches in which DMTs can be utilized. The first approach is the Escalation approach, which involves starting with a lower-potency drug and gradually increasing it if the initial drug fails to control the disease (sub-optimal response). This approach is considered to be a more conservative and safer option, as it allows for the gradual introduction of stronger drugs while monitoring the patient's response and side effects. The second approach is the Induction approach, which involves using a higher potency drug initially to achieve faster control of the disease. This approach is usually reserved for patients with more severe disease or those who are experiencing a relapse. While this approach may provide faster control, it also carries a higher risk of adverse effects and requires closer monitoring. The third approach is the Rescue approach, which involves using a high potency drug to control a relapse or disease flare-up. This approach is typically reserved for patients who have failed to respond to other DMTs or who have more aggressive disease. While this approach can be highly effective in controlling disease activity, it also carries a higher risk of adverse effects and requires close monitoring for potential complications [[25], [26], [27]].
✓ *DMT non-adherence:* Discontinuation of the DMT for more than two months for any reason [28].

### 2.5 Statistical analysis

After collecting the data, it was entered into IBM® SPSS® Statistics version 26. Central and dispersion indices were then used to summarize quantitative variables, while ratios and percentages were used to describe qualitative data. This data was then summarized using tables and graphs. To determine the differences between patients with benign and malignant types of RRMS in various areas, non-parametric statistical tests such as Monte Carlo, Pearson's chi-squared (or Fisher's exact test), and Mann-Whitney *U* Test were utilized. All tests were 2-tailed, and a *P*-value of ≤0.05 was considered statistically significant.

## 3 Results

In this study, a total of 254 patients with RRMS were assessed, out of which 174 patients exhibited the benign type while 80 patients were diagnosed with malignant RRMS.

### 3.1 Demographic differences

Our study revealed that patients with malignant RRMS typically have a higher mean age of onset, as seen in Table 1, suggesting that onset after 30 years may be a negative prognostic factor. However, benign RRMS patients displayed a wide age range at onset, with a significant standard deviation (SD = 17.19) from the mean age of 25.70 years, indicating considerable heterogeneity. We employed the Mann-Whitney *U* Test to accurately compare age of onset between groups, accommodating this variability. The test, detailed in Table 1, resulted in a statistically significant *P.*value of <0.001, confirming that the observed age differences are substantial and not due to random variation.

**Table 1:** Comparison of different parameters among patients with benign & malignant forms of Relapsing-Remitting Multiple Sclerosis.

Parameter		Patients with benign RRMS (N = 174)	Patients with malignant RRMS (N = 80)	*P-*value
Age of onset (Year) (Mean ± SD)		25.70 ± 17.19	32.00 ± 7.96	<0.001^a^
Sex	Male	32 (18.4%)	32 (40.0%)	0.014**
	Female	142 (81.6%)	48 (60.0%)	
Positive familial Hx of MS		40 (23.0%)	14 (17.5%)	0.483**
Number of relapses in the first 5 years (Median; Mean ± SD)		2; 2.10 ± 1.10	4; 3.92 ± 1.56	<0.001^a^
Episodes of hospitalizations in the first 5 years (Median; Mean ± SD)		1; 1.65 ± 1.78	2; 2.48 ± 1.92	0.006^a^
Appropriate DMT adherence in the first 5 years		132 (75.9%)	54 (67.5%)	0.323**

a Mann-Whitney *U* Test, ** Pearson's chi-squared (or Fisher's exact test); Abbreviations: RRMS = Relapsing-Remitting Multiple Sclerosis, SD = Standard Deviation, Hx = History, DMT = Disease-modifying therapy.

Moreover, women had a higher incidence of RRMS in both the benign and malignant groups. Nevertheless, there is a significant difference in the sex distribution between these two groups (*P* = 0.014), with a higher proportion of men suffering from the malignant form of the disease. Specifically, the ratio of men to women among patients with benign RRMS is approximately 2:8, whereas it increases to 4:6 among those with the malignant type (Table 1). Thus, despite a higher frequency of women in both groups, the proportion of men in the malignant group is considerably higher. This indicates that, although RRMS is more prevalent in women, the likelihood of men developing the malignant type of the disease is greater. Consequently, male gender should be regarded as a negative prognostic factor.

The familial history of MS was not significantly different between the two groups; therefore, this study's findings do not support it being considered a prognostic factor (Table 1).

### 3.2 Clinical differences

As demonstrated in Table 1, patients with malignant RRMS experienced a significantly higher number of relapses and hospitalizations during the initial 5 years of treatment.

**Fig. 1:**
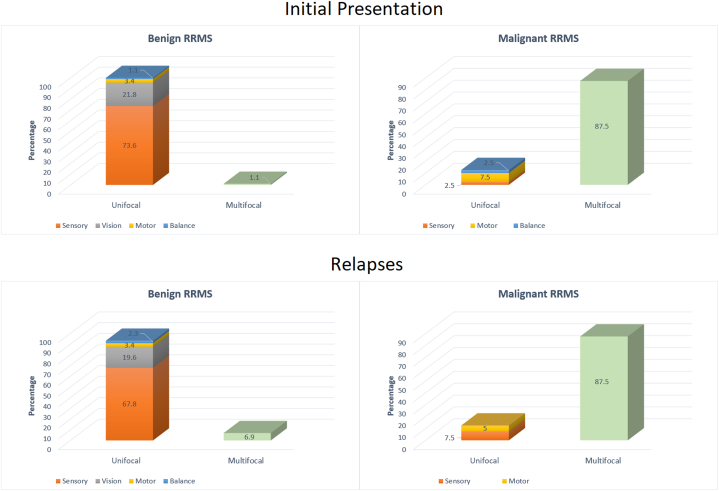
Frequency percentage of different clinical manifestations in the initial presentation and relapses of patients with benign & malignant types of Relapsing-Remitting Multiple Sclerosis; Abbreviations: RRMS = Relapsing-Remitting Multiple Sclerosis.

The clinical manifestations of malignant MS, whether at the time of diagnosis or during relapses, were mainly multifocal involvements, as depicted in Fig. 1. Interestingly, this differs (*P* < 0.001) from the benign form of the disease, where multifocal involvements represented only a small proportion of clinical manifestations, and the unifocal sensory attacks were the most prevalent types of their clinical presentations (Fig. 1).

### 3.3 MRI characteristics

**Fig. 2:**
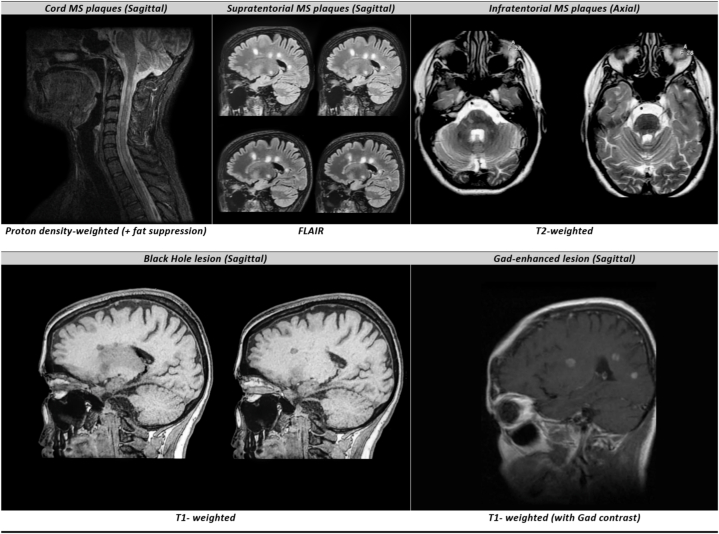
Different types of evaluated MRI plaques and lesions in this study (participants consented to have these images published); Abbreviations: Gad = Gadolinium.

**Fig. 3:**
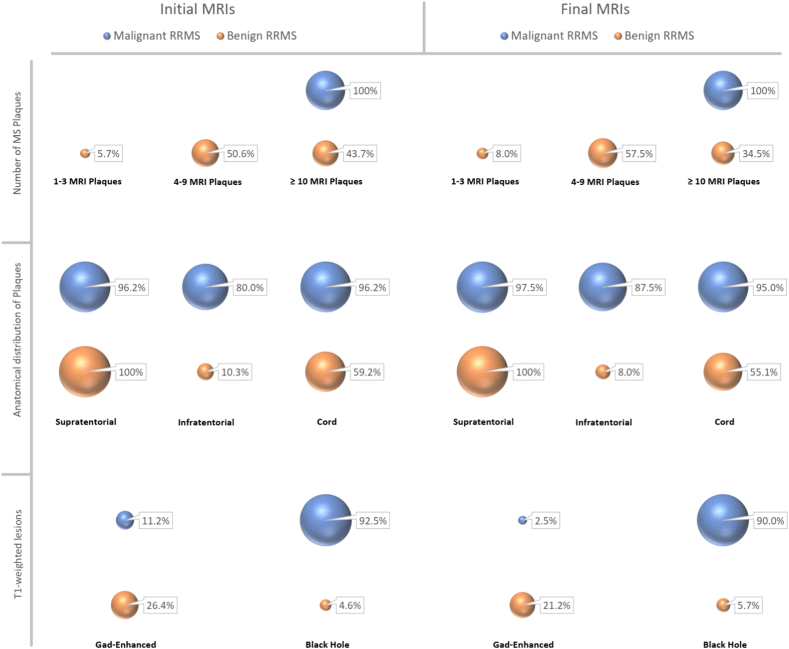
Comparison of MRI characteristics in patients with benign & malignant types of Relapsing-Remitting Multiple Sclerosis; Abbreviations: RRMS = Relapsing-Remitting Multiple Sclerosis.

This study evaluated different types of MRI plaques and lesions, and examples are provided in Fig. 2. The MRI characteristics of patients were compared in Fig. 3, which revealed that the initial and final MRIs of patients (both benign and malignant groups) were generally similar with no significant difference in most cases (also see the Supplemental Tables 1, 2, & 3). Further analysis showed that in patients diagnosed with malignant RRMS, there was a greater load of MS plaques observed. Specifically, all malignant patients had a minimum of 10 plaques, while the majority of benign-type patients had between 4 and 9 plaques. (*P* < 0.001). Also, an anatomical comparison revealed that the number of patients with malignant RRMS having plaques in the infratentorial and cord regions was significantly higher (*P*-values <0.05). Similarly, a higher number of patients in the malignant group had black hole lesions, which represent more severe CNS tissue disruption in these patients (*P* < 0.001).

Detection of new MS lesions is commonly done through enhancements on post-contrast T1-weighted MRI (Gadolinium enhancement) [29]. The percentage of patients with Gad-enhanced lesions in initial MRIs was not significantly different between the benign and malignant groups (*P* = 0.128), even though the benign group had a higher percentage of patients with this lesion. However, evaluating final MRIs followed different results, and this time the number of patients with benign RRMS who had Gad-enhanced lesion was significantly higher (*P* = 0.006).

### 3.4 Therapeutic differences

Table 1 displays the comparison of treatment adherence, indicating that there was no significant difference in this aspect between the two patient groups.

**Fig. 4:**
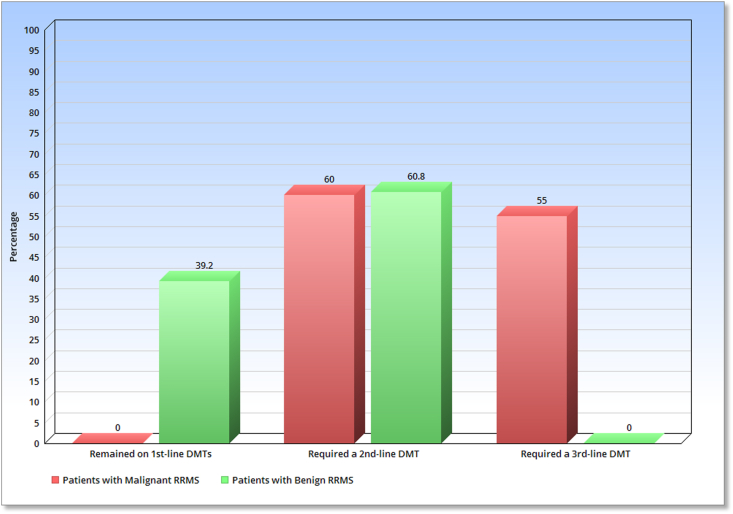
Study's findings on the effectiveness of first-line DMTs and need for second and third-line DMTs in patients with benign & malignant forms of Relapsing-Remitting Multiple Sclerosis; Abbreviations: RRMS = Relapsing-Remitting Multiple Sclerosis; DMT = Disease-modifying therapy.

Table 2 provides a detailed breakdown of the different types of DMT used in the patients under examination. Almost all patients with benign RRMS were treated with a first-line DMT using an escalation strategy, as shown in Table 2. Fig. 4 indicates that over one-third of these patients did not require a change in DMT and showed positive outcomes for at least a decade with the initial DMT. On the other hand, 60.8% of benign RRMS patients needed to upgrade to a higher-level DMT, with Fingolimod being the most common option, followed by Rituximab and Natalizumab (Table 2). The changes in medication resolved their issues, and none of the benign cases required a third-line drug, as depicted in Fig. 4. These findings demonstrate the efficacy of the escalation approach in managing patients with benign RRMS.

**Table 2:** Comparison of the clinical requirement to use different Disease-Modifying Therapies among patients with benign and malignant forms of Relapsing-Remitting Multiple Sclerosis in the studied population.

DMT	Therapeutic line	Patients with benign RRMS		Patients with malignant RRMS	
		As the starting DMT	Total usage	As the starting DMT	Total usage
Interferon	1st	152 (87.3%)	152 (87.3%)	50 (62.5%)	50 (62.5%)
Dimethyl fumarate	1st	8 (4.6%)	8 (4.6%)	0	0
Glatiramer acetate	1st	6 (3.5%)	6 (3.5%)	0	0
Fingolimod	2nd	6 (3.5%)	60 (34.4%)	4 (5%)	8 (10.0%)
Natalizumab	2nd	2 (1.1%)	10 (5.7%)	0	2 (2.5%)
Ocrelizumab	2nd	0	0	0	2 (2.5%)
Rituximab	2nd	0	36 (20.7%)	12 (15%)	36 (45.0%)
Cyclophosphamide/Mitoxantrone	3rd	0	0	14 (17.5%)	44 (55.0%)
Aggregation		174 (100%)	–	80 (100%)	–

Nonetheless, the results for the malignant group were notably different (Table 2). Despite 62.5% of patients in this group starting treatment with a first-line DMT (= escalation approach), none of them remained on this therapy after 5 years (as illustrated in Fig. 4). Furthermore, many patients required last-line therapy, indicating that even the second line of treatment was largely ineffective in this group. In other words, for the majority of malignant patients, escalation was necessary on more than one occasion, suggesting that the efficacy of the escalation approach is limited in managing the disease progression of malignant RRMS. To put it differently, escalation therapy results in a squandering of time and resources for individuals with malignant RRMS and offers inadequate benefits to them. Thus, other approaches such as induction or rescue therapy may be more appropriate in this population. It is important to note that based on our results, we cannot evaluate the efficacy of other approaches, and further research with a larger sample size of malignant RRMS patients is needed to assess the effectiveness of alternative therapeutic strategies.

On a different point of view, the study's findings suggest that when patients with RRMS require multiple changes in DMTs (more than one change), it may be indicative of malignant disease progression and serve as a negative prognostic factor.

## 4 Discussion

The present study found that patients with malignant RRMS had a significantly higher mean age of disease onset, and patients with younger ages at the time of disease onset tend to have a benign form of the RRMS. Moreover, advanced age at the time of disease onset may explain the poor response to first-line DMTs and the need to use higher-line medications in patients with malignant MS. Weidman et al.'s study (2017) [30] supports the notion that the greatest therapeutic effect of DMTs used in MS is before the age of 40. Unfortunately, many patients diagnosed with malignant MS are over 40, which may contribute to the ineffectiveness of the medications used. Also, A study conducted by Malpas et al. (2020) [31] in Australia that aimed to identify clinical markers that could predict the occurrence of severe forms of MS, which found that older age at disease onset is an early indicator of severe MS. Regarding the time to reach EDSS 6 in both malignant and non-malignant groups, Hampshire et al. (2017) [21] conducted a retrospective study in Brazil. The study found that older age at the onset of the disease was more frequent in the malignant group. Furthermore, Menon et al. (2013) [23] conducted a study on patients with MS in Colombia and Canada, revealing that the onset of the disease at an advanced age was significantly associated with the malignant transformation of the disease.

In the current research, out of the 254 patients diagnosed with RRMS, 190 individuals (constituting approximately 75% of the total) were women. Notably, among patients with the benign form of the disease, the percentage of female patients exceeds 80%. However, this proportion drops to 60% among those with the malignant form of RRMS. In essence, these findings suggest that while women may be more susceptible to developing MS, men are more prone to experiencing the malignant type of the disease, which tends to have a poorer prognosis. The higher involvement of the female sex in the pathogenesis and development of MS -as well as many other autoimmune diseases-is a known and significant concern [[32], [33], [34]]. The increased prevalence of MS in females, particularly during puberty [35], implies a potential role of female hormones in the inflammatory, neurodegenerative, and neuroreparative mechanisms that characterize MS [36,37]. Notably, hyperestrogenism has been associated with a favorable outcome in the inflammatory phase, while hyperandrogenism appears to be associated with disease progression [33]. In this regard, many researchers have identified female gender as a favorable prognostic factor [33,[38], [39], [40], [41], [42], [43], [44], [45], [46], [47]]. Koutsis et al. (2010) [48] and Koch et al. (2010) [49] concluded that females need a longer time to reach EDSS 4 and to develop secondary progressive types of MS. Although, a smaller number of studies have not detected any significant gender-based prognostic impact [[50], [51], [52], [53]]. Even, Leibowitz et al. (1970) [54] reported that male sex has a positive prognostic value in patients with RRMS. As the result, despite numerous studies conducted on the prognostic role of gender in patients with RRMS, a comprehensive consensus has yet to be reached. However, the majority of researches in this regard suggested that female gender may serve as a positive predictive factor, a finding that is consistent with the results of this study.

The present study found no statistically significant difference between family history of MS in patients with benign and malignant forms of the disease. A study conducted in Iran by Salehi et al. (2022) [55] on familial MS epidemiology surveyed 9200 patients with MS and found that approximately 19% had a family history of MS. The study did not find a significant relationship between the severity of the disease based on the EDSS scale and family history of MS, which is consistent with the present study's results.

In this recent investigation, it was found that there was no observable distinction in the continuity of treatment between patients with benign and malignant MS. From another point of view, it means that despite consistent medication usage and regular upgraded treatment, still, a significant number of individuals with MS continued to experience a progressive course. In other words, while DMTs may have the potential to prevent an increase in plaque buildup for patients with malignant MS, they do not appear to be effective in altering the disease progression. In this regard, Menzin et al. (2013) [56] discovered that adherence rates to DMT treatment in MS patients ranged from 41% to 88%, and non-adherent patients were at a higher risk of relapse or disease progression, whereas patients who continued DMT had a lower likelihood of hospitalization or emergency room visits. Furthermore, a prospective cohort study by Bawand et al. (2022) [28] revealed that about one in five patients with RRMS did not adhere to treatment during the first five years of treatment. Patients who adhered to DMT had a lower mean EDSS of 0.92 ± 1.09 after five years, compared to non-adherent patients who had a mean EDSS of 1.76 ± 1.17, indicating a significant difference (P-value <0.001) in developed disabilities. As it is clear, the results of present study contradict previous evidence on the effect of DMT non-adherence on the clinical course of patients with RRMS. This may be due to the method of selection of the statistical population, as only patients who fit the definitions of benign and malignant RRMS were studied, and a larger portion of patients who were in the middle of this spectrum was excluded from the study. In other words, it appears that the impact of treatment continuity on patients with benign and malignant MS, two opposite ends of the MS spectrum, is not significant, unlike the majority of the MS population.

The present study observed a significant correlation between MRI findings and MS malignancy. The presence of a higher burden of MS plaques, specifically exceeding 10 plaques, particularly in the infratentorial and cord regions, is associated with disability and an elevated EDSS during the initial 5 years of the disease. Furthermore, the frequency of black holes and negative Gad-enhancement was significantly higher in patients with malignant MS than in benign cases. Tintore et al. (2019) [57] discovered that a higher load of MS lesions at the beginning of the disease was associated with the risk of developing severe MS and cumulative disability. In a separate study by Tintore et al. (2015) [58] identifying demographic, clinical, radiological, and pathological factors affecting disease progression, demographic and topographical factors had a low effect, Oligoclonal band (OCB) had a medium effect, and MRI lesions had a large effect on determining the prognosis of patients with MS. However, based on the results of a review study, Reynders et al. (2017) [59] concluded that the prediction of disease progression based on MRI parameters was limited. Moreover, Sahraian et al.'s (2010) [60] study highlighted that despite the utilization of various detection methods, such as advanced imaging techniques like magnetization transfer imaging and magnetic resonance spectroscopy, the correlation of persistent black holes with clinical outcomes in MS patients remains uncertain. This was inconsistent with our results, whereas Fernández et al.'s (2013) [61] study, in line with the present study, identified the presence of black hole lesions in the MRI of MS patients as a negative prognostic factor.

In the present study, it was observed that patients with malignant MS had a significantly higher frequency of relapse and hospitalization in the first 5 years of the disease as compared to those with benign MS. These findings align with the results of the study of Hampshire et al. (2017) [21], which reported that patients with malignant MS had more attacks and shorter intervals between them than those with benign MS.

### 4.1 Limitations

1. The study design is retrospective and observational, which limits the ability to identify all potential confounding variables.
2. The study only evaluated the efficacy of the escalation approach in managing RRMS patients, and did not assess the effectiveness of induction or rescue therapy.
3. The unavailability of some therapeutic choices, such as Cladribine, Alemtuzumab, Ofatumumab, and stem-cell-based therapies in Iran may limit the study's findings.

## 5 Conclusion

Our study presents a novel view of MS as a spectrum, rather than a singular disease. We highlight that the current practice of treating most MS patients with the same approach may not suit everyone, especially those with a malignant MS phenotype. Our findings suggest that MS encompasses a range from benign cases, showing minimal disability over a decade, to malignant cases with rapid disability progression within five years. The midpoint of this spectrum includes patients with intermediate prognoses. Therefore, it is necessary to review treatment strategies and accurately identify prognostic factors to identify people at higher risk of malignant MS.

The results of the study showed that patients with malignant RRMS had a higher mean age of onset, a higher proportion of men, experienced more relapses and hospitalizations, and showed a greater load of MS plaques in the infratentorial and cord regions with more severe CNS tissue disruption (black hole lesions). Multifocal attacks at the beginning and during the course of the disease were common clinical manifestations of malignant RRMS. The study also found that patients with malignant RRMS had a worse response to the escalation approach of DMTs and required more frequent use of second and last-line therapies.

In contrast, patients with benign RRMS typically exhibit more Gad-enhanced lesions at the onset and throughout the course of the disease, and also had a better response to the escalation approach, and the same initial DMT was used for over a decade, indicating that this approach is a successful treatment strategy for this population.

The study's results may assist clinicians in predicting the prognosis of RRMS patients and making informed decisions regarding their management, ultimately leading to better outcomes for patients with different types of RRMS (Table 3).

**Table 3:** Summary of effective prognostic factors in Relapsing-Remitting Multiple Sclerosis.

	Factor	Associated with good prognosis	Associated with poor prognosis
**Demographic factors**	Age of onset	Lower ages	Higher ages
	Sex	Female	Male
**Clinical factors**	Number of relapses in the first 5 years	Lower	Higher
	Episodes of hospitalizations in the first 5 years	Lower	Higher
	Initial presentation	Unifocal	Multifocal
	Relapses	Unifocal	Multifocal
**MRI-related factors**	Number of MS plaques	<10	≥10
	Anatomical distribution of plaques	Absence of Infratentorial & Cord plaques	Having Infratentorial & Cord plaques
	Black hole lesions in T1-weighted MRI	Absence of black hole lesions	Having black hole lesions
	Gad-enhanced lesions in T1-weighted MRI	Having Gad-enhanced lesions	Absence of Gad-enhanced lesions
**Therapeutic factors**	Type of DMT	Clinical indication for first-line DMTs	Clinical indication for Higher-line DMTs
	Frequency of need to change DMT	0–1	≥2
	Responding to the escalation therapeutic approach	Appropriate	Non-appropriate

Abbreviations: Gad = Gadolinium, MS = Multiple Sclerosis; DMT = Disease-modifying therapy.

## Ethics

All of the research meets the ethics guidelines, including adherence to the national legal requirements. Ethics committee (Code. IR.UMSHA.REC.1400.831) and the Institutional Review Board (IRB) (No. 140011199613) approvals for this study were obtained from the Hamadan University of Medical Sciences. Informed consent according to the Declaration of Helsinki has been obtained from all participants.

## Funding

The authors declared that this study has received no financial support.

## Data availability statement

The data that support the findings of this study are available from the corresponding author upon reasonable request.

## CRediT authorship contribution statement

**Masoud Ghiasian:** Writing – review & editing, Validation, Supervision, Resources, Project administration, Methodology, Conceptualization. **Rashed Bawand:** Writing – review & editing, Writing – original draft, Visualization, Validation, Software, Methodology, Investigation, Formal analysis. **Sulmaz Jabarzadeh:** Writing – original draft, Resources, Methodology, Investigation, Data curation, Conceptualization. **Abbas Moradi:** Writing – original draft, Software, Resources, Formal analysis, Data curation.

## Declaration of competing interest

The authors declare that they have no known competing financial interests or personal relationships that could have appeared to influence the work reported in this paper.
