# Supplementary material for: Predictive factors and treatment challenges in malignant progression of relapsing-remitting multiple sclerosis
Source: Heliyon. 2024 Feb 17;10(4):e26658. doi: 10.1016/j.heliyon.2024.e26658 (PMC10900812; doi:10.1016/j.heliyon.2024.e26658)
Supplement: Multimedia component 1 [file mmc1.pdf]

Supplemental Table 1. Number of MS plaques in the initial and final MRIs

	Initial MRIs plaques				Final MRIs plaques			
	1-3	4-9	≥ 10	*P*.value	1-3	4-9	≥ 10	*P*.value
**Malignant Patients (n = 80)**	0	0	80 (100%)	*< 0.001*	0	0	80 (100%)	*< 0.001*
**Benign Patients (n = 174)**	10 (5.7%)	88 (50.6%)	76 (43.7%)		14 (8.0%)	100 (57.5%)	60 (34.5%)	

Supplemental Table 2. Anatomical distribution of MS plaques in the initial and final MRIs

	Initial MRIs plaques				Final MRIs plaques			
	Supratentorial	Infratentorial	Cord	*P*.value	Supratentorial	Infratentorial	Cord	*P*.value
**Malignant Patients (n = 80)**	77 (96.2%)	64 (80.0%)	77 (96.2%)	*< 0.05*	78 (97.5%)	70 (87.5%)	76 (95.0%)	*< 0.05*
**Benign Patients (n = 174)**	174 (100%)	18 (10.3%)	103 (59.2%)		174 (100%)	14 (8.0%)	96 (55.1%)	

Supplemental Table 3. Number of MS patients with T1-weighted MRI lesions

	Initial MRIs lesions		Final MRIs lesions	
	Gad-enhanced	Black Hole	Gad-enhanced	Black Hole
**Malignant Patients (n = 80)**	9 (11.2%)	74 (92.5%)	2 (2.5%)	72 (90.0%)
**Benign Patients (n = 174)**	46 (26.4%)	8 (4.6%)	37 (21.2%)	10 (5.7%)
P.value	*0.128*	*< 0.05*	*< 0.05*	*< 0.05*
